# Changes of multi-scale structure during mimicked DSC heating reveal the nature of starch gelatinization

**DOI:** 10.1038/srep28271

**Published:** 2016-06-20

**Authors:** Shujun Wang, Xiu Zhang, Shuo Wang, Les Copeland

**Affiliations:** 1 Key Laboratory of Food Nutrition and Safety, Ministry of Education, College of Food Engineering and Biotechnology, Tianjin University of Science & Technology, Tianjin 300457, China; 2 Tianjin Food Safety & Low Carbon Manufacturing Collaborative Innovation Center, Tianjin 300457, China; 3 Plant Breeding Institute, Faculty of Agriculture and Environment, The University of Sydney, NSW 2006, Australia

## Abstract

A thorough understanding of starch gelatinization is extremely important for precise control of starch functional properties for food processing and human nutrition. Here we reveal the molecular mechanism of starch gelatinization by differential scanning calorimetry (DSC) in conjunction with a protocol using the rapid viscosity analyzer (RVA) to generate material for analysis under conditions that simulated the DSC heating profiles. The results from DSC, FTIR, Raman, X-ray diffraction and small angle X-ray scattering (SAXS) analyses all showed that residual structural order remained in starch that was heated to the DSC endotherm end temperature in starch:water mixtures of 0.5 to 4:1 (v/w). We conclude from this study that the DSC endotherm of starch at a water:starch ratio of 2 to 4 (v/w) does not represent complete starch gelatinization. The DSC endotherm of starch involves not only the water uptake and swelling of amorphous regions, but also the melting of starch crystallites.

Starch is a major component of the harvestable parts of many food crops. It occurs as water-insoluble granules, which are composed of two types of α-glucans-amylose and amylopectin, which make up 98–99% of the dry weight. The ratio of the two polysaccharides and their structures vary with the botanical origin of the starch granules. Amylose is mainly located in the amorphous core area of the starch granules, with some interspersed radially among the amylopectin clusters^1^,^2^. Amylopectin is a much larger molecule, and it contributes predominantly to the peripheral crystalline organization of native starch granules^3^,^4^,^5^,^6^,^7^,^8^. Starch granule structure is examined at multiple scales, ranging from the nanometer level for the arrangement of glucosyl units into glucan chains, through the organization of amylopectin outer chains into double helices, crystalline and amorphous lamellae, blocklets, and growth rings, to intact granules of micrometer dimensions^9^,^10^. The multi-scale structure of granules and the changes they undergo during processing are the major determinants of starch functionality for food processing and human nutrition^11^,^12^.

Disruption of the multiple levels of granular organization, as occurs during hydrothermal treatment in food processing, follows hydration, swelling and solubilization of starch molecules, collectively referred to as starch gelatinization^13^,^14^,^15^,^16^,^17^. Various techniques are used to gain new insights into the process of starch gelatinization, including viscosity measurements, optical microscopy, electron microscopy, differential scanning calorimetry (DSC), X-ray diffraction (XRD), small angle X-ray scattering (SAXS), NMR spectroscopy, and FTIR and Raman spectroscopy^18^,^19^. The mechanism of starch gelatinization is studied by DSC in combination with other structural analyses of starch materials prepared under conditions that are always different from those used in DSC measurements. There have been a few studies to characterize the actual changes in starch structure that occur during gelatinization, as represented by DSC endothermic transitions. A combination of DSC with *in situ* structural characterization techniques such as XRD and SAXS has indicated that residual crystallinity and lamellar organization still remain at the completion of the gelatinization endotherm^17^,^20^,^21^,^22^. However, these techniques require specialized equipment and may not be suitable to study of starch gelatinization over a wide range of water contents. Therefore, there is a need for a simpler, more versatile protocol that can provide sufficient material to allow multiple analytical tools to be used to examine simultaneous structural changes of starch during DSC heating over a wide range of water content.

The finding that there is still considerable residual crystallinity and lamellar structure at the end of starch gelatinization endotherm^17^,^20^,^21^,^22^ raises questions about the extent to which starch gelatinization is characterized by DSC and whether the often assumed gelatinization endotherm represents the complete gelatinization of starch granules. In our previous studies^23^,^24^, we investigated the effect of water content on the thermal transitions of different starches over a wide range of water content. The DSC endothermic transition of starch granules at a water:starch ratio of 2:1 or 3:1 was proposed to reflect the limited swelling behaviour of starch rather than complete gelatinization. To test this hypothesis and to further characterize what the DSC endothermic transition of starch denotes, we have designed a protocol using the Rapid Visco Analyser (RVA) to heat starch:water mixtures under conditions that resemble DSC heating as closely as possible. To the best of our knowledge, this is the first time the RVA has been used to simulate DSC heating profiles for the preparation of gelatinized starch samples over a wide range of water content to probe the molecular mechanism underlying gelatinization.

## Experimental Sections

### Materials

Wheat (Zhoumai 18) flour was kindly provided by the Institute of Crop Science, Chinese Academy of Agricultural Science. Amylose (A0512) and amylopectin (A8515) from potato starch were purchased from Sigma Chemical Co. (St. Louis, MO, USA). Other chemical reagents were all of analytical grades. Starch isolation and characterization of its chemical composition are described elsewhere^25^.

### Differential Scanning Calorimetry

Differential scanning calorimetry (DSC) measurements were performed using a Differential Scanning Calorimeter (200F3, Netzsch, Germany) equipped with a thermal analysis data station. An empty aluminum pan was used as the reference. Samples of approximately 3.0 mg of native wheat starch (10.6% moisture) were weighed accurately into an aluminum sample pan. Distilled water was added with a pipette to obtain water:starch ratios (v/w) of 0.5, 0.75, 1.0, 1.5, 2.0, 3.0 and 4.0 in the DSC pans, corresponding to water contents of 33.3, 42.9, 50.0, 60.0, 66.7, 75.0 and 80.0%, respectively. The pans were sealed and allowed to stand for 4 h at room temperature before analysis. The pans were heated from 20 to 95 °C at a heating rate of 10 °C/min. For the freeze-dried starch samples, prepared as described subsequently using an RVA instrument, a ratio of water:starch of 4 (v/w) was used in the DSC measurements.

### Preparation of Starch Samples Using a RVA Instrument

**Figure 1:**
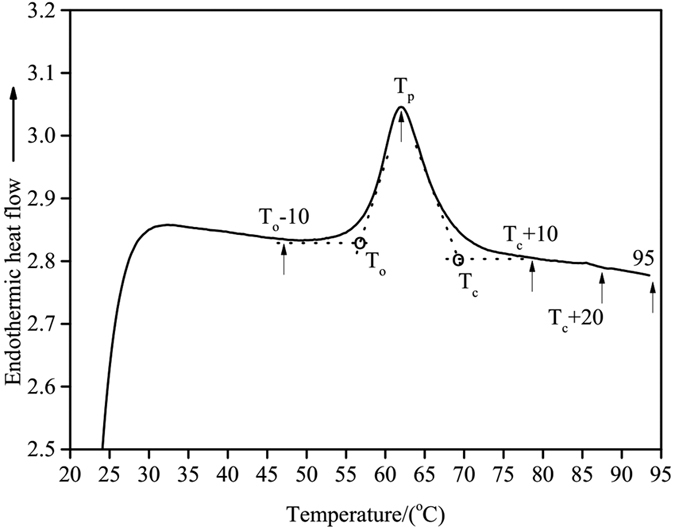
A typical DSC thermogram of a starch-water system with a heating rate of 10 °C/min. The each letter or number shown on the DSC thermogram represented the end temperature of RVA pre-heating.

To obtain sufficient material to characterize structural changes of starch during DSC heating, samples were prepared by heating starch-water systems in a RVA instrument without stirring under the same heating profile described above for DSC measurements. Approximately 2.5 g of native starch (10.6% moisture) were weighed accurately into an RVA canister and distilled water was added with a pipette to obtain the water:starch ratios of 0.5, 0.75, 1.0, 1.5, 2.0, 3.0 and 4.0 (v/w). The mixtures in the canister were agitated gently with a glass rod and allowed to stand for 4 h at room temperature before heating. Each water:starch mixture was heated separately to T_o_-10, T_o_, T_p_, T_c_, T_c_ + 10, T_c_ + 20 and 95 °C with the paddle immobilized (T_o_, T_p_ and T_c_ represent DSC onset, peak and conclusion temperatures, respectively), as shown in Fig. 1. After heating, the samples were freeze-dried, ground into a powder, and passed through a 100 μm sieve. A water:starch (4:1, v/w) mixture that was freeze-dried without prior heating was used as a control.

### FTIR Spectroscopy

The FTIR spectra of freeze-dried starch samples after RVA heating were obtained using a Tensor 27 FTIR spectrometer (Bruker, Germany) equipped with a KBr beam splitter and a DLATGS detector. The spectra were scanned in the range of 4000–400 cm^−1^, with an accumulation of 64 scans and a resolution of 4 cm^−1^. All spectra were automatically baseline-corrected in the range of 1200 and 800 cm^−1^ by OMNIC 8.0 before being deconvoluted with a half-band width of 19 cm^−1^ and an enhancement factor of 1.6. The ratio of absorbances at 1047/1022 cm^−1^ was used to estimate the short-range ordered structure of starch^25^.

### Laser Confocal Micro-Raman (LCM-Raman) Spectroscopy

Raman spectra of freeze-dried starch samples were obtained using a Renishaw Invia Raman microscope system (Renishaw, Gloucestershire, United Kingdom) equipped with a Leica microscope (Leica Biosystems, Wetzlar, Germany). A 785 nm green diode laser source was used in this study^26^. The full width at half maximum (FWHM) of the band at 480 cm^−1^, which can be used to characterize the molecular order of starch, was calculated using the software of WiRE 2.0.

### X-ray Diffraction Analysis

X-ray diffraction analysis was carried out using a D/max-2500kv/pc X-ray diffractometer (D8, Bruker, Germany). All of the starch samples were stored for one week in a desiccator over a saturated NaCl solution, to equilibrate samples to a constant humidity before measurements^27^. The XRD pattern was obtained from 4° to 35° (2θ) at a scanning speed of 4°/min and a step size of 0.02°. The relative crystallinity was quantified as the ratio of the crystalline area to the total area between 4° and 35° (2θ) using the Origin software (Version 8.0, Microcal Inc., Northampton, MA, USA).

### Small-angle X-ray Scattering (SAXS)

Synchrotron SAXS experiments were conducted on the beam line BL16B1 at Shanghai Synchrotron Radiation Facility (SSRF, China). A monochromatic beam of 0.124 nm was used and the sample-to-detector distance was 1860 mm, which provided a *q*-range from 0.11 to 2.25 nm°. The *q* resolution of the instrument at the main lamellar peak at approximately 0.6 nm^−1^ was calculated to be 0.01 nm^−1^. Samples were presented in 2 mm sealed quartz capillaries as suspensions containing excess water above the sedimented sample and scattering was measured for 60 seconds. A sealed 2 mm quartz capillary filled with water was used as a background. SAXS data sets were averaged radially using Bruker AXS software 4.1.30. SAXS curves were normalized to sample transmission and background-subtracted using fit 2D.

### Field-Emission Scanning Electron Microscopy (FE-SEM)

The freeze-dried samples were mounted on a stub with double-sided adhesive tape, sputter-coated with gold before imaging using a field-emission scanning electron microscope (1530, LEO, Germany). An accelerating voltage of 5 kV was used during imaging.

### Statistical Analysis

Results are reported as the mean values and standard deviations of at least duplicate measurements. Analyses of variance (ANOVA) by Duncan’s test (p < 0.05) were conducted using the SPSS 19.0 Statistical Software Program (SPSS Inc. Chicago, IL, USA).

## Results And Discussion

### Effect of Water Content on Thermal Properties of Native Wheat Starch

The effect of water content on thermal transition parameters of wheat starch is presented in Table 1. All the starch-water systems showed the typical DSC endothermic transition between 55 and 70 °C, with well-defined onset (T_o_), peak (T_p_) and conclusion (T_c_) transition points (thermograms not shown). The values for T_o_, T_p_ and T_c_ increased from 55.8 to 57.5 °C, from 60.3 to 62.0 °C and from 63.8 to 67.4 °C, respectively, with increasing water content in the mixture, although the increase was not statistically significant in some cases. Enthalpy change (ΔH) increased from 1.8 to 9.6 J/g as the water/starch ratio increased from 0.5 to 2, above which ΔH remained essentially constant with further increases in water content. Similar results for thermal transition of starch as a function of water content were also reported in many previous studies^23^,^24^,^28^,^29^,^30^. The endothermic transition of starch granules was proposed previously to represent both the water absorption and swelling behaviour of starch granules, with the end point of the endotherm G corresponding to the completion of water absorption into granules^23^,^24^. As water content increases, so does the potential for water absorption into granules, leading to higher T_o_, T_p_ and T_c_ and greater ΔH due to increasing disruption of starch crystallites.

### Thermal Properties of Starches Pre-heated in the RVA

**Figure 2:**
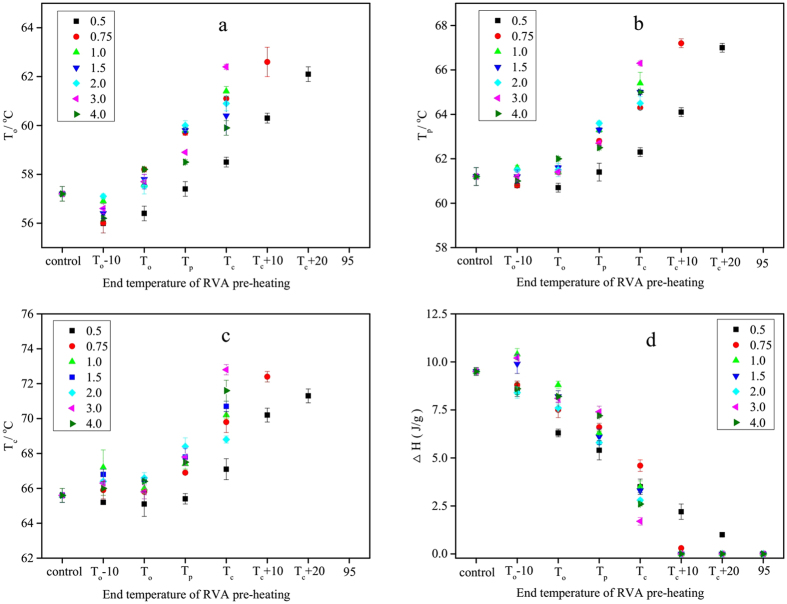
Thermal transition parameters of starch samples after pre-heating to different temperatures in RVA canisters. (**a**) T_o_ of starch samples; (**b**) T_p_ of starch samples; (**c**) T_c_ of starch samples; (**d**) ΔH of starch samples.

Starch samples were pre-heated in the RVA to the designated temperatures (Fig. 1) in different starch:water mixtures before measuring the DSC thermal transition parameters. A typical DSC endothermic transition was observed for all pre-heated starch samples in a starch-water mixture of 1:0.5, with the exception of starch pre-heated to 95 °C, which did not present an endothermic transition. At a starch:water ratio of 1:0.75, no endothermic transition was observed only for starch samples pre-heated to T_c_ + 20 and 95 °C. However, no endothermic transitions were observed for starch:water mixtures of 1:1 or greater pre-heated to T_c_ + 10 or higher. In general, T_o_, T_p_ and T_c_ increased with increasing end temperature of RVA pre-heating (Fig. 2a–c). Two possible explanations, not mutually exclusive, are proposed for this observation. Firstly, less stable starch crystallites have already melted during the initial RVA pre-heating, and hence the melting of more stable crystallites that remain require greater energy input, leading to higher transition temperatures during the DSC protocol. Secondly, pre-heating of the starch-water systems resulted in disruption of some starch crystallites thereby increasing the amount of amorphous regions in starch^31^. As water preferentially enters the amorphous regions of starch granules, the ingress of water is prolonged^32^, leading to higher T_o_, T_p_ and T_c_ of starch crystallites not disrupted during the pre-heating phase.

In contrast, ΔH of all starch samples decreased noticeably with increasing end temperature of RVA pre-heating, indicating the gradual disruption of starch crystallites. The value of ΔH after pre-heating to T_c_ decreased from 5.0 to 1.7 J/g as the water/starch ratio increased from 0.5 to 4.0, and interestingly, declined further in samples that were pre-heated to higher temperatures (Fig. 2d). With RVA pre-heating to T_c_ + 10, ΔH was barely measurable with the exception of the water:starch mixture of 0.5:1, indicating that complete disruption of starch crystallites had occurred after RVA pre-heating to this temperature.

### Short-range Ordered Structure of Starch Samples Determined by FTIR and LCM-Raman Spectroscopy

**Figure 3:**
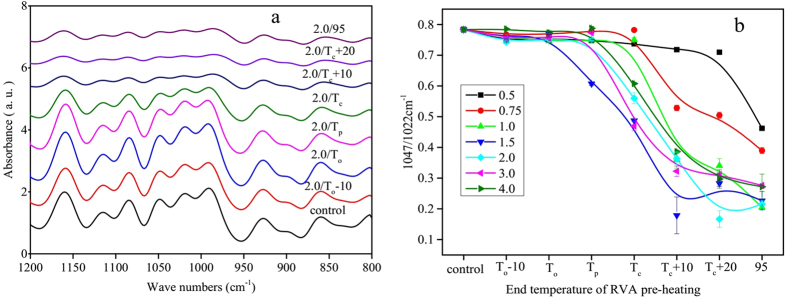
The FTIR spectra (**a**) and ratios of absorbances at 1047/1022 cm^−1^ (**b**) of starch samples.

The loss of short-range molecular order of double helices during DSC heating can be monitored by FTIR and Raman spectroscopy^25^. The IR bands around 1047 and 1022 cm^−1^ are sensitive to changes in the crystalline and amorphous regions of starch, and the ratio of absorbances at 1047/1022 cm^−1^ is often used as a measure of the amount of ordered structure in starch. As all of the FTIR spectra of starch samples were similar, only the deconvoluted FTIR spectra in the range of 1200 to 800 cm^−1^ of the 2:1 water:starch mixture are presented (Fig. 3a). The corresponding ratios of absorbances at 1047/1022 cm^−1^ are shown in Fig. 3b. In general, the intensity of IR bands decreased with increasing end temperature of RVA pre-heating. The ratios of absorbances at 1047/1022 cm^−1^ remained essentially constant with increasing end temperature of RVA pre-heating below T_p_, but decreased significantly above T_p_. The ratios of absorbances at 1047/1022 cm^−1^ continued to decrease in samples pre-heated above T_c_, indicating that residual short-range ordered structure was still present. At low water:starch ratios, the ratios of absorbances at 1047/1022 cm^−1^ decreased much more slowly than values obtained from starch samples at high water:starch ratios.

**Figure 4:**
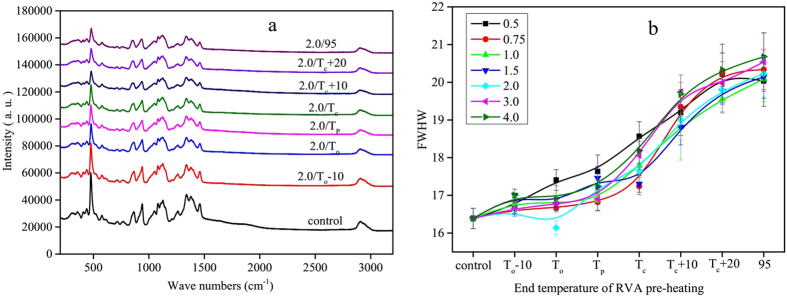
The LCM-Raman spectra (**a**) and FWHM values (**b**) of starch samples.

As all of the starch samples presented similar LCM-Raman spectra, only those of 2:1 water:starch mixtures are presented (Fig. 4). Several clear bands can be seen at 480, 865, 943, 1264 and 2900 cm^−1^, which are related to δ (CH2), νs (C1-O-C4), νs (C1-O-C5), skeletal (C-C-O), and ν (C-H) modes, respectively^33^. Of these, the bands at 480 and 2900 cm^−1^ are sensitive to changes of ordered structure in starch^23^,^24^,^33^,^34^. The full width at half maximum (FWHM) of the most intense band at 480 cm^−1^ is strongly and positively related to the relative crystallinity of starch^25^,^33^. The intensity of the Raman bands decreased progressively with increasing end temperature of RVA pre-heating, with the largest change occurring in the band at 480 cm^−1^. The FWHM of the various water:starch mixtures increased with increasing end temperature of RVA pre-heating. Similar to the FTIR results, the FWHM did not change greatly with RVA pre-heating below T_p_, but increased substantially above T_p_. The FWHM continued to increase with increasing end temperature of RVA pre-heating above T_c_, showing that short-range ordered structure still remained in pre-heated starch samples, consistent with the FTIR results.

### Long-range Ordered Structure of Starch samples Determined by XRD

**Figure 5:**
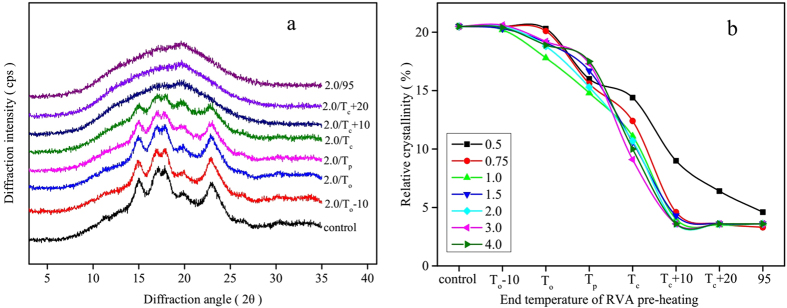
The XRD diffraction patterns (**a**) and relative crystallinity (**b**) of starch samples.

As all of the starch:water mixtures presented similar XRD patterns, only the diffractograms of water:starch ratio of 2:1 are shown (Fig. 5a). Native wheat starch presented a characteristic A-type X-ray diffraction pattern with four clear peaks occurring at 15, 17, 18 and 23° (2θ). An additional weak peak at 20° (2θ) was attributed to the amylose-lipid complex. On pre-heating to temperatures up to T_c_, all starch samples showed the typical A-type X-ray diffraction patterns, although the peak intensities decreased with increasing end temperature of RVA pre-heating. With pre-heating to T_c_ + 10 and above, the disappearance of diffraction peaks indicated the starch crystallites were almost completely disrupted. The relative crystallinity only decreased sharply with pre-heating to a temperature above T_p_. Starch samples at various water contents after RVA pre-heating to T_c_ still presented a clearly discernible X-ray diffraction pattern, with residual crystallinity ranging from 10 to 14.4% (Fig. 5b). This observation was consistent with the DSC, FTIR and Raman results, which all showed a residual ordered structure of starch samples at various water contents after pre-heating to T_c_.

### Lamellar Structure Measured by SAXS

**Figure 6:**
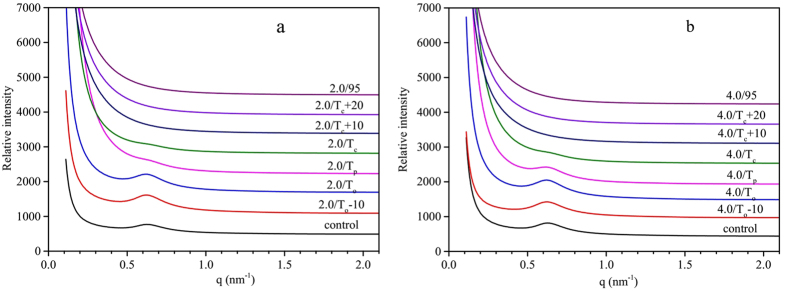
The SAXS pattern of starch samples at a starch:water ratio of = 1:2 (**a**) and 1:4 (**b**).

As the SAXS patterns of all starch samples were similar, only the patterns of samples at water:starch ratios of 2:1 and 4:1 are presented (Fig. 6). The SAXS pattern of native wheat starch was characterized by intense scattering at low scattering vector (*q*), which rapidly decreased at larger angles and featured a well-resolved peak around *q* of 0.60 nm^−1^. This peak was assumed to arise from the periodic arrangement of alternating crystalline and amorphous lamellae, corresponding to lamellar *d*-spacing of 10.0 nm^35^.The lamellar peak intensity increased with end temperature of RVA pre-heating to T_o_, above which it decreased gradually. The initial increase in lamellar peak intensity indicated that the density contrast between crystalline and amorphous lamellae increased initially with increasing end temperature of RVA pre-heating. As starch is heated at a temperature below T_o_, annealing may occur, leading to the improved quality of starch crystallites^25^. This may account for the increased intensity of the lamellar peak of starch after pre-heating to T_o_-10 and T_o_. For starch samples that were pre-heated to a temperature up to T_p_, the characteristic lamellar peak was clearly identified and its *q* value was essentially unchanged indicating the lamellar *d*-spacing of starch samples remained almost constant. With pre-heating temperature increasing beyond T_p_, the lamellar peak intensity decreased up to T_c_, indicating residual lamellar structure was still present in starch samples after pre-heating to this temperature. The peak essentially disappeared with pre-heating above T_c_. Taking these results together, we can conclude that the lamellar structure of starch was little affected after pre-heating to T_p_, above which it was severely disrupted.

### Granular Morphology of Starch Samples

**Figure 7:**
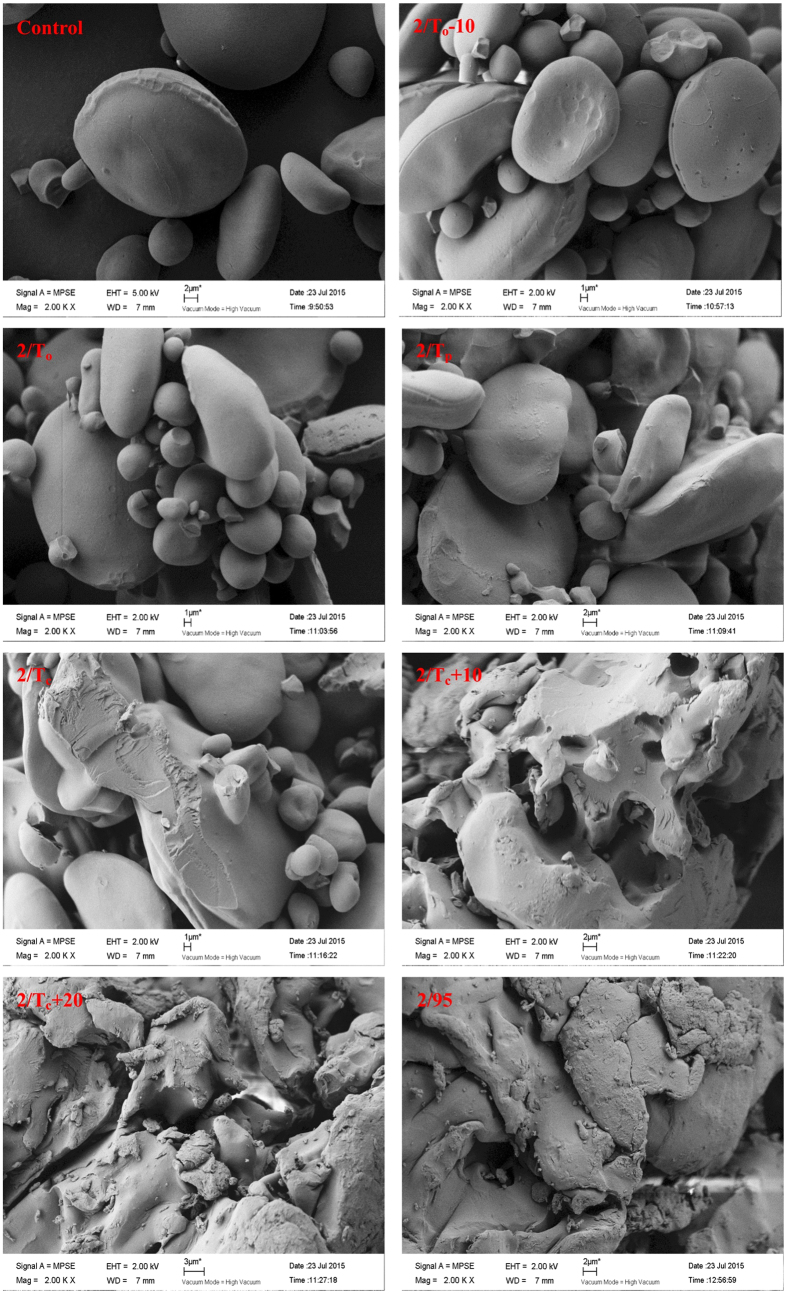
SEM images of wheat starch samples after preheating to different temperatures, starch:water = 1:2. Magnification, 2000x.

SEM micrographs of starch samples at a water:starch ratio of 2:1 after pre-heating are shown in Fig. 7. No significant differences were noted in granular morphology between native starch and starch samples after pre-heating to a temperature up to T_p_. After pre-heating to T_p_, some starch granules were deformed and adhered as a result of small granule swelling followed by freeze-drying. Many starch granules were still clearly observed for all starch samples after pre-heating to T_c_ (Fig. 7 2.0/T_c_). These observations were generally consistent with the previous reports^23^,^24^.

## General Discussion

A key finding of the present study was that at all water:starch ratios studied, some structural order was still present at the end of DSC endothermic transition (i.e., T_c_), and that further disruption occurred on further heating beyond T_c_. At low to intermediate water content (water:starch ratio < 2:1), the DSC endothermic transition is considered to represent partial gelatinization of starch due to limited swelling and melting of starch granules^23^,^24^. This partial gelatinization was substantiated in the present study by only a partial loss of starch structure after pre-heating to T_c_. At a water:starch ratio of around 2:1 or higher, the DSC endotherm remained essentially unchanged, leading to the constant gelatinization parameters (Table 1). This constant endotherm has long been assumed to represent the complete gelatinization behavior of starch^14^. However, the present study has shown that ordered structures and morphology of starch are still present at T_c_ of the starch gelatinization endotherm at these high water contents, indicating that the constant DSC endotherm obtained at a water:ratio of 2:1 or higher does not represent complete gelatinization of starch. Residual crystallinity, lamellar structure and granular morphology have been reported at T_c_ of the DSC endotherm of starch at a water content of 60% or 66%^17^,^21^,^22^,^23^,^24^.The results of the present study, together with the previous findings, show clearly that the DSC endotherm obtained at water:starch ratios used in many experiments (2:1 to 4:1) do not represent the complete gelatinization behavior of starch.

A close examination of the parameters for monitoring changes in the order of structure of starch during DSC heating indicates that at the end of the starch gelatinization endotherm, loss of long-range crystallinity occurred to a greater extent than that of short-range molecular order. Long-range crystallinity, as measured by XRD, was almost completely lost after heating to T_c_ + 10, except for the starch:water mixture of 0.5. The residual crystallinity of about 4% may be attributed to the amylose-lipid complex naturally present in wheat starch^26^. In contrast, loss of short-range molecular order, as monitored by FTIR and Raman spectroscopy, still occurred in starch pre-heated to T_c_ + 10 and beyond. These results indicate that short-range molecular order continued to be lost beyond T_c_ up to 95 °C, whereas disruption of a substantial amount of the long-range crystallinity occurred within the gelatinization endotherm. The changes in ΔH of starch seemed to coincide more closely with loss of long-range crystallinity than with short-range molecular order. The loss of long-range crystallinity before the disruption of short-range molecular order of starch during heating has been reported in other studies^36^,^37^. This finding is not consistent with a proposal that enthalpy change of starch reflects primarily the loss of double helical order, based on a study in which the samples analysed were prepared under different conditions to those used for DSC measurements^16^. However, our findings are consistent with the proposal that the initial entry of water into the amorphous regions transmits disruptive forces via the long-range connecting structures into the crystalline regions^17^.

Another interesting result from the present study is that when starch was pre-heated up to the temperature of T_p_, only a small amount of starch structure was lost, indicating that the heat input during this phase of DSC heating is used mainly for the uptake and movement of water into amorphous regions of starch granules. The substantial disruption of starch structure occurred mostly at the later stage of DSC endotherm, which could be due to the cooperative melting of starch crystallites. These results can lead us to conclude that DSC gelatinization endotherm involves not only the initial water uptake and swelling of amorphous regions in starch granules (within T_p_), but also the melting of starch crystallites (beyond T_p_).

## Conclusions

Changes in the molecular order of starch during the DSC gelatinization endotherm were investigated for a wide range of water:starch mixtures that were pre-heated in a RVA to simulate the DSC heating profiles. Multiple analyses of starch samples prepared under conditions that mimicked the DSC heating profile showed that the starch still contained appreciable amounts of long- and short-range ordered structure at the conclusion temperature of the endotherm over water:starch ratios ranging from 0.5 to 4.0. The loss of long-range crystallinity appeared to be greater than that of short-range molecular order in the DSC endotherm to T_c_. We conclude that the gelatinization endotherm of starch does not represent complete starch gelatinization, and reflects more the loss of long-range crystallinity than short-range molecular order. The gelatinization endotherm involves not only the water uptake and swelling of amorphous regions, but also the melting of starch crystallites (beyond T_p_).

## Additional Information

**How to cite this article**: Wang, S. *et al*. Changes of multi-scale structure during mimicked DSC heating reveal the nature of starch gelatinization. *Sci. Rep.* **6**, 28271; doi: 10.1038/srep28271 (2016).

## Figures and Tables

**Table 1:** Thermal transition parameters of native wheat starch.

Water:Starch (v/w)	Water content (%)	T_o_ (^o^C)	T_p_ (^o^C)	T_c_ (^o^C)	ΔH (J/g)
0.5	33.3	55.8 ± 0.2c	60.3 ± 0.6de	63.8 ± 0.1c	1.8 ± 0.0e
0.75	42.9	55.3 ± 0.3d	60.8 ± 0.1cd	65.4 ± 0.2b	2.7 ± 0.1d
1.0	50.0	55.7 ± 0.0cd	60.0 ± 0.2e	64.9 ± 0.4bc	5.7 ± 0.1c
1.5	60.0	56.8 ± 0.21b	61.0 ± 0.1c	65.3 ± 0.1b	7.2 ± 0.3b
2.0	66.7	57.0 ± 0.1ab	61.4 ± 0.1bc	65.6 ± 0.1b	9.6 ± 0.2a
3.0	75.0	57.4 ± 0.1a	61.8 ± 0.2ab	67.5 ± 0.4a	10.0 ± 0.5a
4.0	80.0	57.5 ± 0.2a	62.0 ± 0.2a	67.4 ± 0.5a	9.8 ± 0.0a

Values are means ± SD. Values with the same letters in the same column are not significantly different (p < 0.05).
